# Personalized medicine: Has it started yet? A reconstruction of the early history

**DOI:** 10.3389/fgene.2012.00313

**Published:** 2013-01-08

**Authors:** Frank Emmert-Streib

**Affiliations:** Computational Biology and Machine Learning Laboratory, Center for Cancer Research and Cell Biology, School of Medicine, Dentistry and Biomedical Sciences, Faculty of Medicine, Health and Life Sciences, Queen's University BelfastBelfast, UK

**Keywords:** personalized medicine, genomics, high-throughput data, translational medicine, biomedical informatics

## Abstract

Within the last few years the field personalized medicine entered the stage. Accompanied with great hopes and expectations it is believed that this field may have the potential to revolutionize medical and clinical care by utilizing genomics information about the individual patients themselves. In this paper, we reconstruct the early footprints of personalized medicine as reflected by information retrieved from PubMed and Google Scholar. That means we are providing a data-driven perspective of this field to estimate its current status and potential problems.

## Introduction

The Human Genome Project (Lander et al., 2001; Venter et al., 2001; Consortium, 2004) did not only result in the first sequencing of the human DNA but it also fostered technological advances exploitable beyond its initial purpose (Quackenbush, 2011). As a result from such technologies a variety of different types of “Omics” data (Ghosh and Poisson, 2009; Moreno-Risueno et al., 2010), e.g., genomics, transcriptomics, proteomics, metabolomics, and epigenomics data (Lee et al., 2002; Förster et al., 2003; Rual et al., 2005; Stelzl et al., 2005; Palsson, 2006; Sechi, 2007; Garbett et al., 2008; Yu et al., 2008) can nowadays be generated to measure molecular entities on all relevant biological levels. With respect to a medical application these technologies generated an immediate impetus on the field *personalized medicine* because it aims to integrate genomics and clinical data from individual patients in order to improve patient care by identifying more efficient treatment strategies (Auffray et al., 2009; Ginsburg and Willard, 2009; Hamburg and Collins, 2010; Fernald et al., 2011).

In this paper we present a data-driven overview of the early history of personalized medicine. We are using information retrieved from PubMed and Google Scholar to obtain quantitative information statistics of published articles and the community structure of scientists that share a common interest in this topic. From this information we try to identify the potential impact personalized medicine has made so far but also to reveal indicators of problems that might prevent its blossoming. Despite the fact that PubMed and Google Scholar provide a wealth of curated data about various aspects of published articles over the last decades and the research interests of thousands of scholars it should be emphasized that neither has been designed to serve the purpose of our analysis. For this reasons, the drawn conclusions should only be seen as an indicator. Nevertheless, utilizing databases like PubMed and Google Scholar allows to present arguments beyond a mere opinion due to the possibility to compare different aspects quantitatively with each other.

Specifically, to allow for a simplified interpretation of the retrieved numbers from PubMed and Google Scholar we present whenever appropriate a comparison with related topics. For example, rather than presenting an interpretation based on absolute numbers of, e.g., published research papers and review or editorial papers published for personalized medicine we perform a similar analysis for related fields like bioinformatics and systems biology and present a comparative interpretation. This relieves us from the burden to introduce to many *ad hoc* assumptions that may lead to erroneous interpretations if they would be too far-off the truth.

## Current state of personalized medicine

In order to obtain a general overview of the current state of the field *personalized medicine* we extract information from the literature. Specifically, we are using information provided by PubMed and compare publications in personalized medicine to other fields. Further, we investigate also the content of such publications, as far as this information is provided by PubMed.

For our following analysis we used the tool FLink provided by the NCBI. FLink allows to perform a conventional PubMed search with the additional feature that the results can be downloaded as a csv file. The obtained csv files are than parsed by scripts we wrote in the statistical programming language R. The advantage of this approach is that we have convenient access to the same information that is visually represented by a conventional PubMed search but can process this information as desired. For the following analysis we retrieved all data in November 2012.

**Figure 1:**
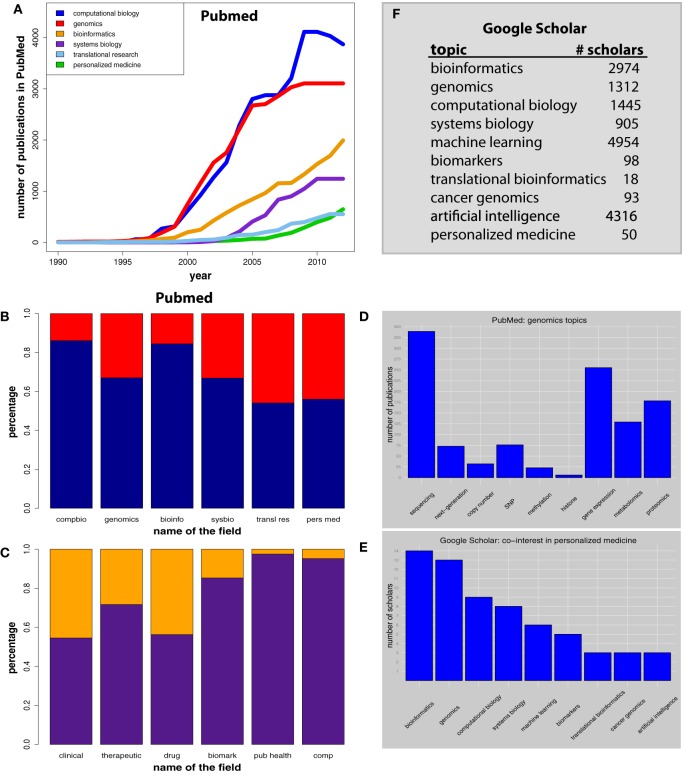
**(A)** Number of PubMed publications. **(B)** Distribution of publications of the two categories “research paper” (blue) and “non-research paper” (red). **(C)** Distribution for conjugated searches. Orange corresponds to publications containing the search terms “personalized medicine” and “topic” (on the x-axis) and purple corresponds to all remaining publications. **(D)** Distribution of genomics data types, shown on the x-axis. **(E)** People interested in personalized medicine are also interested in the topics on the x-axis. **(F)** Numbers of scholar interested in the listed topics.

We start by showing in Figure 1A the number of publications per year in computational biology (blue), genomics (red), bioinformatics (orange), systems biology (purple), translational research (sky blue), and personalized medicine (green). We selected these fields because they are closely related to personalized medicine. From these curves one can see that personalized medicine emerged very recent in the literature becoming notable only around 2005, which makes it the newest of all shown fields with a steep increase within the recent 5 years. More established fields like computational biology and genomics seem to have peaked already by having reached their maxima.

Next, we try to get an insight into the distribution of research papers and non-research papers from the listed PubMed publications. Specifically, we define as “non-research” papers review, comment and editorial articles and as research papers we define all remaining articles. Figure 1B shows the distributions over these two categories for the same six fields as in Figure 1A, namely, computational biology (compbio), genomics, bioinformatics (bioinfo), systems biology (sysbio), translational research (transl res), and personalized medicine (pers med).

The red proportion in each bar corresponds to the percentage of publications that are non-research papers, whereas the blue proportion corresponds to all other publication types. From a comparison of the results one finds that in translational research and in personalized medicine there is a larger percentage of publications devoted to strategic and conceptual questions as represented by review articles, comments or editorial papers and a smaller proportion of publications corresponds actually to original research papers. This could be a reflection of the novelty of these fields and the hopes that accompanies them. For this reason there may be a higher proportion of articles that try to pave the way for these fields, instead of actually contributing new results either from experimental or computational work.

In general, personalized medicine has been conceived as a way to improve medical and clinical patient care by taking into account patient specific data. Hence, personalized medicine is inherently connected to a clinical application. In Figure 1C, we show the results of conjugated PubMed searches by querying “personalized medicine” and one additional keyword, as given on the x-axis of this Figure. From left to right: clinical, therapeutic, drug, biomarkers (biomark), public health (pub health), and computational (comp). In Figure 1C the orange proportion corresponds to the publications that contain both search terms, whereas the purple proportion corresponds to all remaining publications. The first three keywords (clinical, therapeutic, and drug) are frequently present, as could be expected from the purpose of personalized medicine. In contrast, the term “public health” is not frequently present. This is plausible because personalized medicine is on the level of an individual of a population, whereas public health investigates the population as a whole.

It is somewhat surprising to see that the terms “biomarkers” and “computational” are not frequently present either. This is surprising because personalized medicine is data-driven and these data are analyzed by computational methods aiming to identify important signatures, e.g., in form of biomarkers. A possible reason for the underrepresentation of these terms could be again given by the novelty of this field, as discussed for Figure 1A. Another explanation could be the underappreciation of computational methods. That means these methods could be used, however, without appropriately describing them in the methods section of publications which would almost inevitably require the authors to use the term “computational.” Third, there could be even another reason for this. Specifically, there could be experimental studies that conduct genetics or genomics experiments without actually conducting research one would identify as personalized medicine, but instead, the authors connect their results to this field either in the introduction or the conclusion section of a publication. Unfortunately, PubMed does not provide information about the individual sections of a publication that would allow to zoom-in in more detail.

## Diversity of utilized genomics data

In order to obtain a general overview of the used genomics data types that occur in any publication type, including original research, review and editorial articles, related to personalized medicine, we use again PubMed and search for conjugated hits, i.e., “personalized medicine” and “term,” whereas the queries for “term” correspond to the x-axis in Figure 1D. More precisely, to capture a similar meaning of slightly different terms we also include variations of these terms. For instance we count publications that include “next generation” as well as “next-generation.”

It is not surprising that “sequencing” surmounts all other terms because it is the prototype technology to obtain genetic information about various aspects of the DNA (Mardis et al., 2008; Shendure and Ji, 2008; Ansorge, 2009). More surprising is to see that “gene expression” and “proteomics” appear also quite frequently in publications. It is reassuring to see this trend because the DNA represents only static information about the state of a cell whereas transcriptomics and proteomics data are capable of reflecting the dynamic state of molecular activity. For this reason it is likely that information about the DNA alone is not sufficient to accomplish the noble goals of personalized medicine.

## Community structure of personalized medicine

Due to the multidisciplinarity of personalized medicine it would be interesting to know what other interests people have that are interested in personalized medicine. In order to answer this question we are using Google Scholar.

In Figure 1E we show a histogram over fields that are listed on the Google Scholar pages of scientists that have an interest in personalized medicine. Specifically, at the time of the analysis there were 50 people that used the term “personalized medicine” to indicate their interests. From parsing the Google Scholar pages of these 50 individuals by using an R script we developed, we determined the frequency of further interests listed by these scholars. For example, among the 50 people interested in personalized medicine, 14 are also interested in bioinformatics and 8 in systems biology.

Figure 1E shows only the top 9 research interests of the 50 individuals. In total we found 95 different interest terms that have been listed. There are three interesting observations that follow from this figure. First, among the top 9 fields are many “computational” disciplines like bioinformatics, computational biology, or machine learning. This supports the intuitive understanding of personalized medicine described above that computational methods are a key means to approach this field practically. Second, the dominance of computational methods in the top ranked fields implies an underrepresentation of experimental fields as represented by genomics or cancer genomics. Interestingly, looking further down the line to lower ranked research interests we find a more balanced distribution of computational and experimental interests including, e.g., molecular genetics, medical oncology, whole genome sequencing, or clinical trials. Hence, overall individuals interested in personalized medicine come from both directions. Third, comparing the number of scholars we found for personalized medicine with that of other subjects, as shown in Figure 1F, we see that the community of scientists subscribing to personalized medicine is rather small. This corresponds to the results in Figure 1A indicating that the field is barely beyond its infancy.

## Conclusion

It is without a doubt that the principle idea behind personalized medicine holds a great potential for translational medicine by improving diagnostic, prognostic and therapeutic approaches for patient care. However, besides the seminal work of Chen et al. (2012) only relatively few studies have been conducted in a similar manner.

A principal hurdle for a wider and active engagement of the community in this research area is certainly provided by the still considerable costs incurred by the needed patient-based high-throughput experiments. This is despite the steadily declining costs for DNA sequencing. More important, I think that the major problem is a lack of a precise definition of personalized medicine that would allow an efficient experimental design translating the paradigm into practice.

Overall, based on the information we retrieved from PubMed and Google Scholar we think that it is fair to say that personalized medicine is still at the very beginning. It is increasingly recognized within the literature, but a wider impact hasn't been achieved yet.

### Conflict of interest statement

The author declares that the research was conducted in the absence of any commercial or financial relationships that could be construed as a potential conflict of interest.
